# Indirect serum biomarkers perform sub optimally in screening for significant liver fibrosis among HIV-infected and uninfected adults in Uganda

**DOI:** 10.4314/ahs.v22i3.45

**Published:** 2022-09

**Authors:** Clara Wekesa, Rosalind Parkes-Ratanshi, Gregory D Kirk, Jim Aizire, Ponsiano Ocama

**Affiliations:** 1 Infectious Diseases Institute, Makerere University Kampala Uganda; 2 Cambridge University, Institute of Public Health, Cambridge UK; 3 John Hopkins University, Baltimore USA; 4 Makerere University, College of Health Sciences Kampala Uganda

**Keywords:** Liver fibrosis, serum bio-markers, Fibroscan®, HIV/AIDS, Sub Saharan Africa

## Abstract

**Introduction:**

Indirect serum bio-markers present an acceptable noninvasive and cheap alternative for screening of significant liver fibrosis (SLF). Evaluation of their use in resource limited settings is important to determine their utility.

**Methods:**

We conducted a cross sectional study among 520 HIV infected and HIV uninfected adults attending care clinics in Kampala Uganda. Presence of SLF was determined using Fibroscan® liver stiffness measurement of ≥7.2KPa. The diagostic value of indirect serum bio-markers for diagnosis of SLF was evaluated using the area under the receiver operating characteristics curve (AUROC) using Fibroscan® as gold standard.

**Results:**

Overall AUROC values for Age Platelet Index (API), Aspartate to Alanine Ratio (AAR), AST-to-Platelet Ratio Index (APRI), Fibrosis Index based on 4 Factors (FIB-4) and Gamma glutamyl transferase to Platelet Ratio Index (GPR) were 0.52, 0.49, 0.55, 0.55 and 0.54 respectively. Among HIV-infected participants AUROC values were slightly improved at predicting presence of SLF but still under 70%.

**Conclusion:**

Despite APRI and FIB-4 being more likely to identify participants with SLF, the overall diagnostic value of all serum bio-markers was poor with and without stratification by HIV status. We recommend the use of Fibroscan® technology as more accurate non-invasive diagnostic method for screening of SLF.

## Introduction

Mortality and morbidity resulting from significant liver fibrosis (SLF) is becoming more prevalent^1^, ^2^. Regardless of the cause, chronic liver injury leads to SLF which is the primary risk factor for complications, such as cirrhosis and liver cancer^1^, ^3^. Significant liver fibrosis occurs when there is excess deposition of extracellular matrix by the stellate cells of the liver as a response to chronic injury^2^,^4^. As changes alter the structure and or function of the liver, assessment of the liver structure and function is used to clinically detect and gauge the extent of liver fibrosis. Evaluation of SLF is critical in clinical practice to prognosticate patients, make treatment choices, as well as monitor response to therapy and disease progression^3^–^5^. The gold standard evaluation for SLF traditionally is liver biopsy, but it is limited by sampling and observational error^6^, ^7^. In addition, it requires special expertise to be performed and for pathological specimen to be read; all of which limit its use within resource limited settings. By virtual of its invasive nature, it is not readily acceptable by patients^7^.

Recent advancements in diagnostic methods for SLF advocate for the use of non-invasive techniques. Non-invasive techniques for diagnosis of SLF are broadly categorized as imaging or blood based techniques, some of which usually have standardized interpretation requiring less technical expertise. Due to the ease of their administration and reduced patient risk they can be performed repetitively, an advantage in monitoring disease progression and response to therapy^8^.

Imaging techniques include transient elastograpy (Fibroscan ®) which has been shown to be comparable in performance to liver biopsy for most liver disease conditions^5^,^8^, ^9^. The volume of the liver explored by Fibroscan® is at least 100 times more than that explored by liver biopsy and results are immediate^10^. However Fibroscan® machines remain costly for most low and middle income countries which limits their use to research settings rather than widespread clinical use^11^. Indirect serum bio-markers present a more feasible option given that they are derived from cheap and routinely performed tests and standardized indices are easily interpretable by non-specialized clinical personnel aiding decentralized use. 7, 8, 12. Given the limitations in use of liver biopsy and or Fibroscan ® in low resource settings, indirect serum bio-marker indices may suffice as the next available option to screen for SLF to facilitate early referral and management of liver disease.

Early screening purposes to prevent and halt progression of SLF to complicated forms of liver disease for which tertiary management is very costly and most times out of reach. In addition, within the sub Saharan African region, the limited studies on the validation of these indirect serum biomarkers in an African population, have evaluated their use in single disease conditions, mainly viral hepatitis, and their remains disparity in their performance. There is strong recommendation for more similar studies in the region to inform on threshold values for these indirect serum bio-markers when applied to various liver disease entities^11^, ^13^. Furthermore, available recommendations for the use of indirect serum bio-markers by World Health Organization in locations with limited access to the Fibroscan® mainly focuses on use of APRI among persons with chronic hepatitis B yet there is an increase in risk factor profile for chronic liver disease in the region presently for which the use of APRI may not necessary be suited.

We therefore conducted a cross sectional study to determine the diagnostic value and level of agreement for the diagnosis of SLF for five specified indirect serum bio-markers (API, APRI, AAR GPR and FIB-4) using Fibroscan® as gold standard among adults attending care in urban Uganda.

## Methods

### Study design and population

We conducted a cross sectional study between January 2015 and March 2020 involving 260 HIV-infected and 260 HIV-uninfected adults attending out-patient clinics within the National referral hospital in Kampala. The HIV-infected and HIV-uninfected patients were identified from the Adults Infectious Diseases Clinic (AIDC) and Ear Nose and Throat (ENT) out-patient clinics respectively. The AIDC is a model center of excellence for the care and management of HIV-infected adults. The prevalence of chronic hepatitis B among patients attending the AIDC is estimated at 11% and sero-prevalence of hepatitis C at 2%. The description of the site and prevalence of other risk factors for liver disease such as tobacco and alcohol use is well described elsewhere^14^–^16^. The ENT clinic is government supported sub specialized surgical out-patient clinic that receives stable patients seeking related services mainly as self-referrals as well as consultative referrals within Mulago national referral hospital, Kampala, Uganda.

### Participant enrolment

#### HIV-infected participant enrolment

We sampled the HIV-infected participants from already collected data from a prospective study, the HIV and Hepatocellular carcinoma in Uganda (H2U) at the AIDC, a study that aims to determine the prevalence and correlates of pre-malignant liver cirrhosis. Using a randomly generated computer list, we selected 260 HIV-infected study participants from a pool of 2000 participants in the H2U database. We included participants with records of liver stiffness measurement (LSM) done by Fibroscan®. Pregnant women and patients with medical implants were excluded from having Fibroscan done.

#### HIV-uninfected participants

Using compiled information on age and gender of the HIV-infected participants from the AIDC, we used these demographic characteristics to enroll the HIV-uninfected participants at the ENT clinic, matched by gender and age group. We accessed a daily clinic register on attendance, identified and approached potential participants requesting initial consent to participate in the study and screen for HIV serology in accordance with the national guidelines^17^. Patients that tested HIV sero-negative were then taken through detailed informed consent. Adults who had no known history of chronic liver disease were eligible to participate in the study. Pregnant women and persons with implanted medical devices were excluded from participation.

### Study Procedures

#### Questionnaire

The information collected at both clinics included sociodemographic information (age, gender); lifestyle habits (on self-reported use of alcohol, tobacco and herbal medicines); anthropometric data (weight and height) and clinical information (viral hepatitis serology, biochemical data, ART related information and drug history).

#### Physical measurements

All anthropometric measurements were done using Seca 761 mechanical scale to the nearest 1kg and a Seca Leicester stadiometer to the nearest 0.1 cm for weight and height respectively.

Laboratory testing: All blood testing was done at the Central laboratory at the Infectious Disease Institute in the Makerere University John Hopkins University (MUJHU) laboratory, a CAP certified laboratory. Complete blood count was done using a Beckman Coulter AcT 5diff analyzer and liver biochemistry was analyzed using HitachiCobas C311. Hepatitis B serology was performed using an enzyme immunoassay (Monolisa HBsAg Ultra 3; Bio-Rad). Hepatitis C antibody testing was done using 3^rd^ generation enzyme immunoassay (Bio-rad Monolisa Anti-HCV PLUS).

Transient elastography (Fibroscan®): Liver stiffness measurements (LSM) for participants from both clinics were done using Fibroscan® (Echosens, Paris France) and the median result of ten Fibroscan® readings with the IQR <30% and accuracy of 60% and above was considered as the final result11. At the time of enrolment for the HIV-infected participants under the H2U study, the only available Fibroscan® probe was the M-probe. In contrast during the enrolment of the HIV-uninfected participants from the ENT clinic the Fibroscan® was upgraded to have both an M and XL probe. The XL probe being suitable for LSM in persons with body mass index (BMI) ≥30 and the M-probe being suitable for persons with BMI less than 30. Participants were not asked to fast prior to the Fibroscan® procedure.

### Computing the indirect serum biomarker indices

Using parameters from the liver biochemistry and platelet count, indices were computed for the selected serum bio-markers (table 1). The indirect serum bio-markers included the API, AAR, APRI, FIB-4 and GPR. The outcome SLF was assessed as a binary outcome for both Fibroscan® and the selected indirect serum bio-markers. On Fibrosca® SLF was defined as having a LSM of >7.1KPa18. The upper limit of normal for aspartate transaminase (AST) and gamma glutamyl transferase (GGT) used were 32 U/L and 55 U/L respectively.

**Table 1:** Summary of serum bio-marker constitution and cut off values for significant liver fibrosis

Bio-marker Index	Markers		Cut-off value for significant fibrosis
Age-Platelet Index (API)	Age, platelets	(10^9^/l): 225 = 0; 200–224 = 1; 175–199 = 2; 150–174 = 3; 125–149 = 4; <125 = 5; Age (years) <30 = 0; 30–39 = 1; 40–49 = 2; 50–59 = 3; 60–69 = 4; 70 = 5	≥6 19, 20
AST to ALT ratio (AAR)	AST, ALT	AST/ALT	≥1^7^, ^19^
AST –Platelet Ratio Index APRI	AST, Platelets	AST level (U/L) / upper normal limit of AST (U/L) x 100 Thrombocyte (109/L)	≥0.7^7^
Fibrosis 4- Score (FIB-4)	Age, Platelets, ALT, AST	((Age expressed in years) × (ASTU/L)) ((PLT109/L)×(ALTU/L1/2))	≥1.45^11^
GGT to Platelet Ratio GPRI	GGT, Platelets	(GGTIU/L/ULN of GGT)/platelet count (10^9^/L) × 100^24^	≥0.32^11^

### Data Analyses

For the descriptive statistics, continuous variables were presented as means and medians. Categorical data were described as proportions and comparisons between two proportions done using chi square test. The diagnostic value of API, AAR, APRI, FIB-4, GPR and Fibroscan ® was evaluated using receiver operating characteristic (ROC) analysis. The sensitivity (Se), specificity (Sp), positive predictive value (PPV), negative predictive value (NPV), diagnostic accuracy (DA), positive likelihood ratio (LR+) and negative likelihood ratio (LR-) were calculated based on the cut-off points as mentioned above. Significance was set at p-value <0.05. Definition of the diagnostic value of the indirect serum bio-markers was as follows; excellent for AUROC value ≥0.9, good for AUROC value 0.80–0.89, moderate for 0.70–0.79 and poor for AUROC value <0.70.

Agreement testing for categorical data comparing two raters was done for Fibroscan® and each indirect serum bio-marker. Cohen's Kappa statistic for agreement testing was used where the prevalence of SLF was ≥10% for each rater. In instances where the prevalence of SLF was lower than 10% for anyone rater, a prevalence adjusted bias adjusted kappa was applied. Interpretation of the Kappa statistic was done as shown below (table 2)^21^. Four of the HIV negative participants were excluded from the analyses because of an invalid LSM.

**Table 2:** Interpretation of Kappa statistic

Value of Kappa	Level of Agreement
0–0.20	None
0.21–0.39	Minimal
0.40–0.59	Weak
0.60–0.79	Moderate
0.80–0.90	Strong
>0.90	Almost perfect

Analyses were performed using STATA 16 statistical package

## Results

There were more female participants in the study (56% vs 44%). The mean age was 44 years (±10.3) and this was similar between both groups of participants. HIV-uninfected participants had a higher mean BMI compared to the HIV-infected participants (27 Vs 23; p<0.001). The overall sero-prevalence of hepatitis C was 3% and this was similar in both groups. Chronic hepatitis B was five times more prevalent among the HIV-infected participants (14% vs 3%; p<0.001). HIV-uninfected participants were more likely to have used herbal drug medicines (49% vs25%; p<0.001), however there were no differences in the use of alcohol and tobacco products. HIV-infected participants had a higher mean serum alanine transaminase (24mmol/l vs 21mmol/l; p0.011). The mean length of ART use among HIV-infected participants was 10 years and 90% had viral suppression. Detail on these general characteristics of the entire study population is provided in Table 3.

**Table 3:** Baseline characteristics of HIV-infected and non HIV-infected participants attending outpatient clinics at Mulago Hospital between 2015–2020 (N=516)

Variable	Total	HIV negative	HIV positive	p-value
	N=516	n=256	n=260	
Sex				0.945
Female	287 (55.6)	142 (55.5)	145 (55.8)	
Male	229 (44.4)	114 (44.5)	115 (44.2)	
Age, mean(sd)	44.6 (10.3)	44.4 (10.2)	44.9 (10.3)	0.577
Body Mass Index, mean(sd) (n=512)	24.9 (8.3)	26.7 (10.6)	23.1 (4.5)	**0.000**
HCV sero-positive(n=511)				0.776
Negative	496 (97.1)	246 (96.9)	250 (97.3)	
Positive	15 (2.9)	8 (3.1)	7 (2.7)	
Hepatitis B surface Antigen (n=511)				
Negative	468 (91.6)	247 (97.2)	221 (86.0)	**0.000**
Positive	43 (8.4)	7 (2.8)	36 (14.0)	
Alcohol consumption (n=509)				0.493
No	294 (57.8)	140 (56.2)	154 (59.2)	
Yes	215 (42.2)	109 (43.8)	106 (40.8)	
Tobacco use (n=509)				0.649
No	427 (83.9)	207 (84.6)	220 (84.6)	
Yes	82 (16.1)	40 (15.4)	40 (15.4)	
Herbs use (n=430)				**0.000**
No	281 (65.4)	86 (50.6)	195 (75.0)	
Yes	149 (34.6)	84 (49.4)	65 (25.0)	
ART duration (n=199)			10.7 (3.0)	
Median HIV viral load (copies/ml)(IQR)			19 (19–74)	
Median ALT (U/L) (IQR)	18(14–26)	17(13–24)	20(14–26)	**0.011**
Median AST (U/L) (IQR)	22(18–27)	21(17–26)	23(18–29)	0.081
Median Platelet count (U/L) (IQR)	250(207–292)	254(213–291)	244(199–295)	**0.055**
Median GGT (U/L) (IQR)	30(19–55)	23(17–37)	41(24–80)	**<0.001**

Overall, of 99 participants were identified with SLF based on Fibroscan®; API identified 13(13%), AAR identified 71 (72%), APRI identified 12(12%), FIB4 identified 24 (24%) and GPR identified 14 (14%) (table 4). Out of 412 participants identified without SLF on Fibroscan®; API picked out 372 (90%), AAR picked out 107 (26%), APRI picked out 392 (95%), FIB4 picked out 344 (83%) and GPR picked out 264 (64%). Overall APRI showed the best diagnostic value with sensitivity of 12% specificity of 97%, PPV of 48%, NPV of 81% and diagnostic accuracy of 80%. AAR had the least diagnostic value with sensitivity of 72%, specificity of 26%, PPV of 19%, NPV of 79% and diagnostic accuracy of 35%.

**Table 4:** Diagnostic value of indirect serum bio-markers versus Fibroscan® in diagnosing significant liver fibrosis among adults attending out-patient clinics at Mulago Hospital

Serum Bio-marker /Cut off	Fibroscan® output		Sensitivity (%)	Specificity (%)	Positive Predictive Value PPV (%)	Negative Predictive Value NNP (%)	Likelihood Ratio		Accuracy
	No fibrosis n=412	Significant Fibrosis n=99					+	-	
**API**									
No Fibrosis n=458	372	86	13.1	91.9	28.3	81.2	1.61	0.95	76.4
Significant Fibrosis n=46	33	13							
**AAR**									
No Fibrosis n=135	107	28	71.7	26.2	19	79.3	0.97	1.1	35
Significant Fibrosis n=373	302	71							
APRI									
No Fibrosis n=479	392	87	12.1	96.8	48	81.8	3.8	0.91	80.2
Significant Fibrosis n=25	13	12							
**FIB-4**									
No Fibrosis n=419	344	75	24.2	84.9	28.2	82.1	1.61	0.89	73.1
Significant Fibrosis n=85	61	24							
**GPR**									
No Fibrosis n=322	264	58	38.3	69.5	23.7	82	1.25	0.89	63.3
Significant Fibrosis n=152	116	36							

When stratified by HIV status, APRI still had the best performance with even better diagnostic value when used among the HIV-infected participants (table 5). The diagnostic accuracy of all serum bio-markers except AAR and GPR was reduced when evaluated within the HIV uninfected participants.

**Table 5:** Diagnostic value of indirect serum bio-markers versus Fibroscan® in diagnosing significant liver fibrosis among HIV infected adults attending out-patient clinic at Mulago Hospital

Serum Biomarker /Cut off	Fibroscan® output		Sensitivity (%)	Specificity (%)	Positive Predictive Value PPV (%)	Negative Predictive Value NNP (%)	Likelihood Ratio		Accuracy (%)
	No fibrosis n=219	Significant Fibrosis n=37					+	-	
**API**									
No Fibrosis n=225	196	29	21.6	91.6	30.8	87.1	2.57	0.86	81.3
Significant Fibrosis n=26	18	8							
**AAR**									
No Fibrosis n=68	57	11	70.3	26.2	13.9	83.8	0.95	1.14	32.6
Significant Fibrosis n=187	161	26							
**APRI**									
No Fibrosis n=238	208	30	18.9	97.2	53.8	87.4	6.75	0.83	85.7
Significant Fibrosis n=13	6	7							
**FIB-4**									
No Fibrosis n=203	177	26	29.7	82.7	22.9	81.8	1.7	0.85	74.9
Significant Fibrosis n=48	37	11							
**GPR**									
No Fibrosis (<0.32) n=131	117	14	61.1	58.8	21.2	89.3	1.48	0.66	59.2
Significant Fibrosis (≥0.32) n=104	82	22							

**Figure 1:**
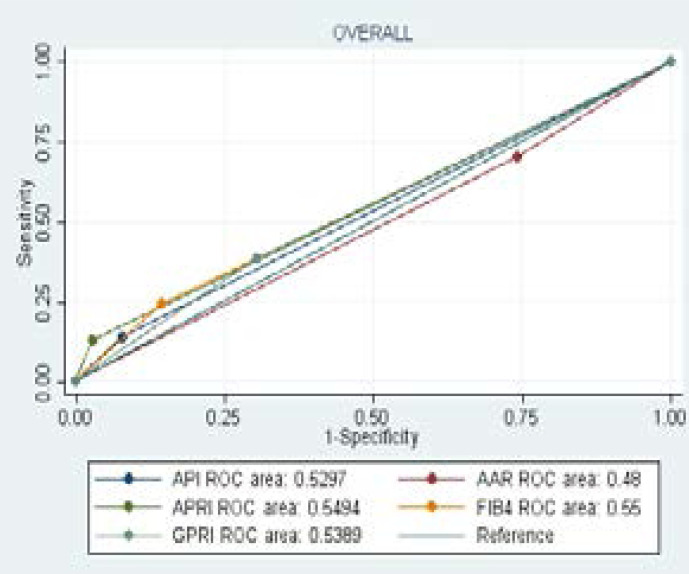
AUROC analysis of indirect serum bio-markers in predicting significant liver fibrosis in the overall population

From the ROC analysis, all 4 serum biomarkers (API, AAR, APRI, FIB-4 and GPR) demonstrated poor diagnostic value even when stratified by HIV-status (AUROC< 0.7) (table 6 and figure 1). The overall AUROC values were as follows; API 0.52 (CI 0.49–0.56 p0.175), AAR 0.49 (CI 0.44–0.54 p0.674), APRI 0.55 (CI 0.51–0.58 p0.009), FIB-4 0.55 (CI 0.50–0.59 p0.050) and GPR 0.54 (CI 0.48–0.59 p0.163). There was slight improvement in AUROC values from the overall estimates on assessment of performance of serum bio-markers when applied to the HIV-infected participants.

**Table 6:** Diagnostic performance and level of agreement between indirect serum bio-markers and Fibroscan® in diagnosing significant liver fibrosis among HIV infected and uninfected adults attending out-patient clinics

Overall						
Model vs Fibroscan	AUROC	95% CI	p-value	Kappa	p-value	Accuracy (%)
API	0.525	0.488–0.561	0.175	0.063	0.061	76.4
AAR	0.489	0.439–0.539	0.674	-0.01	0.666	35.0
APRI	0.545	0.511–0.578	0.009	0.124	0.0001	80.2
FIB-4	0.546	0.500–0.592	0.050	0.970	0.014	73.0
GPR	0.539	0.484–0.593	0.163	0.063	0.074	63.3
**HIV positive**						
API	0.566	0.496–0.635	0.064	0.151	0.0074	81.3
AAR	0.482	0.402–0.562	0.661	-0.013	0.676	32.6
APRI	0.581	0.516–0.645	0.015	0.220	0.000	85.7
FIB-4	0.562	0.483–0.641	0.122	0.111	0.0378	74.9
GPR	0.599	0.512–0.687	0.026	0.112	0.013	59.2
**HIV negative**						
API	0.501	0.462–0.540	0.958	0.003	0.479	71.5
AAR	0.494	0.429–0.558	0.850	-0.007	0.576	37.6
APRI	0.522	0.485–0.558	0.240	0.0605	0.078	74.7
FIB-4	0.542	0.486–0.598	0.143	0.097	0.0519	71.2
GPR	0.527	0.464–0.589	0.401	0.057	0.188	67.4

## Discussion

Overall all the indirect serum bio-markers (API, AAR, APRI, FIB-4 and GPR) had poor diagnostic value in predicting the presence of SLF having AUROC values less than 0.7 even when stratified by HIV status. Indirect serum bio-markers that demonstrated better diagnostic value overall included APRI and FIB-4 and similar among the HIV-infected participants. None of the indirect serum bio-markers significantly performed well among the HIV-uninfected participants. Our findings are similar to other studies in the region conducted among patients with chronic hepatitis B infection that also found low ability to predict presence of SLF using APRI but good performance of GPR^18^, ^22^. The selection of study participants for the latter studies excluded those with conditions known to alter the serum GGT levels, such as alcohol consumption, co-infection with HIV/AIDS and HCV. Our inclusion of these categories of participants may have affected the accuracy of GPR^23^. In addition, other studies seem to suggest that there is poor correlation between GPR and Fibroscan® for evaluation of SLF among HBV/HIV patients in sub Saharan Africa and this may further explained its poor diagnostic performance among our HIV-infected participants^23^. Our findings were also similar to studies conducted among western populations focused on alcoholic liver disease that showed APRI to have poor diagnostic value predicting SLF^24^.

In contrast, other studies still within the region conducted among treatment naïve patients with chronic hepatitis B infection and patients with chronic hepatitis C infection showed APRI, FIB-4 and GPR to have moderate to good diagnostic value in predicting SLF^11^. We had low sample sizes for patients with HBV and HCV mono-infection and this could have possibly affected our estimates. In addition, we screened for HCV using antibody testing whose presence does not necessarily indicate active infection.

We found a high negative predictive value and low positive predictive value for all the indirect serum bio-markers similar to other studies^11^, ^13^, ^18^. For all the serum bio-markers the inter-observer agreement with Fibroscan® for the diagnosis of significant liver fibrosis was poor and for some almost by chance.

## Limitations

Our study had a number of limitations that included, restraints in finances that could not allow for liver biopsy to be used as gold standard. We also did not take fasting LSM that could have led to an over estimation of the burden of SLF. Our study recruited participants from a tertiary health care unit which may not be representative of the general population. We had a limited total number of persons identified with cirrhosis on Fibroscan® (cutoff LSM≥13KPa) and were unable to evaluate the performance of the indirect serum bio-markers for diagnosis of liver cirrhosis to compare with other studies in the region. Screening for hepatitis C was done using serology which often gives false positive results.

Despite these limitations, our study evaluates a number of indirect serum bio-markers in the background of an interplay of several risk factors (that often never exist in isolation) for SLF including HIV infection, which is prevalent in the region. Our study adds to the existent evidence on the disparity of the performance of indirect serum bio-markers when applied in our setting for the evaluation of SLF.

## Conclusion

The indirect serum bio-markers (API, AAR, APRI, FIB-4 and GPR) had unsatisfactory performance in identifying participants with SLF among HIV-infected and uninfected adults in urban Uganda. Given their poor diagnostic value and varied performance, we recommend Fibroscan ® be prioritized as a more accurate non-invasive technique for screening of SLF. We also recommend further studies evaluating the value of repeated testing using indirect serum bio-markers as part of a screening algorithm and/or alternate applications such as in disease prognostication.

## Abbreviations

AAR: Aspartate transaminase to Alanine transaminase Ratio
AIDC: Adults Infectious Disease Clinic
AIDS: Acquired Immunodeficiency Syndrome
API: Age Platelet Index
APRI: Aspartate transaminase to Platelet Index
ART: Anti-retroviral therapy
AST: Aspartate Transaminase
AUROC: Area under the Receiver Operating Curve
BMI: Body Mass Index
ENT: Ear Nose and Throat
FIB-4: Fibrosis-4 Index
GGT: Gamma Glutamyl Transferase
GPR: Gamma Glutamyl Transferase to Platelet index
HBV: Hepatitis B infection
HCV: Hepatitis C infection
HIV: Human Immunodeficiency Virus
IQR: Interquartile range
LR: Likelihood Ratio
LSM: Liver Stiffness Measurement
MUJHU: Makerere University John Hopkins University
NPV: Negative Predictive Value
PPV: Positive Predictive Value
ROC: Receiver Operating Curve
Se: Sensitivity
Sp: Specificity

## Declarations

### Availability of data

Please contact the author for data requests.
